# Consumptive hypothyroidism in an Egyptian baby with benign neonatal hemangiomatosis: a case report

**DOI:** 10.1186/1752-1947-7-48

**Published:** 2013-02-18

**Authors:** Kotb Abbass Metwalley, Hekma Saad Farghaly

**Affiliations:** 1 Pediatric Endocrinology Unit, Department of Pediatrics, Faculty of Medicine, Assiut University, Assiut, Egypt

## Abstract

**Introduction:**

Benign neonatal hemangiomatosis is a condition in which multiple cutaneous hemangiomas appear at birth or shortly thereafter; visceral complications are absent. Here, we report a case of a consumption hypothyroidism in an Egyptian baby with benign neonatal hemangiomatosis.

**Case presentation:**

An 8-month-old Egyptian boy with benign neonatal hemangiomatosis was referred to our institution for evaluation of developmental delay. Initial examination revealed a quiet baby who was able to sit only with support. He had hypotonia, a large anterior fontanelle*,* puffy eyes, cold extremities, hypothermia, bradycardia, and abdominal distension. An examination of his skin revealed more than 100 dome-shaped red-purple cutaneous hemangiomas that varied in size from 5 to 10mm on the back, the abdomen and the extremities without mucus membrane involvement. He had low serum free thyroxine concentration and triiodothyronine levels and high thyroid-stimulating hormone and reverse-triiodothyronine levels. A work-up that involved appropriate imaging ruled out visceral involvement. Based on the above mentioned data, a diagnosis of consumptive hypothyroidism due to benign neonatal hemangiomatosis was made. He was started on oral thyroid medication which was gradually increased to 90μg L-thyroxine daily (15μg/kg/day). After three months of treatment, he was able to sit alone without support and he had normal levels of thyroid-stimulating hormone and serum free thyroxine.

**Conclusion:**

Thyroid function should be assessed periodically in babies with benign neonatal hemangiomatosis, especially if symptoms of hypothyroidism appear or the size and number of hemangiomatosis increase rapidly. Moreover, high doses of L-thyroxine may be needed to achieve euthyroidism during the infancy.

## Introduction

Benign neonatal hemangiomatosis (BNH) is a rare, self-limited disease where multiple capillary hemangiomas occur exclusively in the skin. Spontaneous regression occurs usually within the first two years of life [1]. BNH must be distinguished from diffuse neonatal hemangiomatosis (DNH), a severe condition with a similar cutaneous presentation but with the presence of life-threatening visceral hemangiomas [2]. Consumption hypothyroidism develops when the rate of inactivation of thyroid hormones surpasses the rate of their production and was described in association with DNH in children [3]. Reviewing the literature revealed that hypothyroidism in association with BNH has been reported in only one case [4]. Here, we report a case of a consumption hypothyroidism in an Egyptian baby with BNH.

## Case presentation

**Figure 1:**
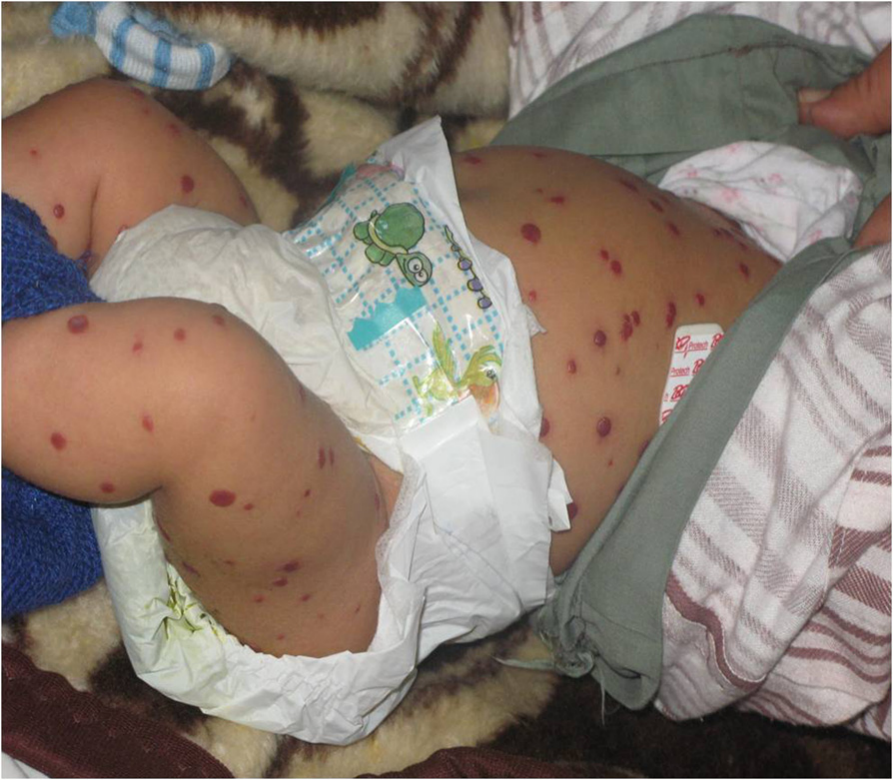
An Egyptian baby with hypothyroidism associated with benign neonatal hemangiomatosis.

An 8-month-old Egyptian boy with BNH was referred to our institution from the Dermatology department for evaluation of poor activity and developmental delay. He was the second child of consanguineous parents; he was delivered at term by Caesarean section following an uncomplicated pregnancy and cried immediately after delivery with a birth weight of 3.5kg. There was no family history of hemangiomas or congenital malformation. Neonatal screening tests were normal. His mother observed the appearance of multiple pinpoint erythematous lesions all over his body at the age of 35 days that increased rapidly in number and size. She reported that her baby remained well until the age of three months*.* At the age of six months, he was unable to roll over prone to supine or vice versa. An examination revealed a quiet baby who was able to sit only with support but unable to orient to voice. His height and weight were 25^th^ percentile. Vital signs on admission were recorded as follows: temperature, 36°C; blood pressure, 80/60mmHg; and heart rate, regular at 66 beats/minute. A physical examination found generalized hypotonia, a wide anterior fontanelle, puffy eyes, cold extremities, and mild abdominal distension. There were no signs of rickets or thyroid enlargement. An examination of his skin revealed more than 100 dome-shaped red-purple cutaneous hemangiomas that varied in size from 5 to 10mm on the back, the abdomen and the extremities with no mucus membranes involvement (Figure 1). Other systematic examinations were unremarkable. Thyroid ultrasonography and scan, ophthalmic examination, laryngoscopy, bone screening and computed tomography (CT) scan of the brain were normal. Repeated chest radiography, echocardiography, and abdominal ultrasonography with a Doppler study were normal which ruled out respiratory system, cardiovascular and hepatic involvement. Laboratory studies revealed that serum electrolytes, hemogram, blood glucose and aminotransferase levels were normal. A hormonal assay revealed a serum thyroid-stimulating hormone (TSH) of 176μIU/mL (normal 0.3 to 5.0μIU/mL), serum free thyroxine (FT4) of 0.4ng/dL (normal 1.0 to 2.5ng/dL), and serum triiodothyronine (T3) of 65ng/dL (normal 85 to 250ng/dL). Serum reverse-T3 (rT3) level was 865ng/dL (normal 10 to 50ng/dL). The mother's thyroid function tests were normal and no thyroid autoantibodies were found in the mother or in the baby. Based on these findings, a diagnosis of consumptive hypothyroidism due to BNH was made. He was started on oral thyroid medication which was gradually increased to 90μg L-thyroxine daily (15μg/kg/day). After one month of treatment, blood hormone levels were: TSH 45mIU/mL, FT4 0.99ng/dL, T3 80ng/dL and rT3 70ng/dL. Blood hormone levels were completely normal two months later (TSH 1.99mIU/mL, FT4 1.44ng/dL and T3 40.23ng/dL) with spontaneous involution of some of his cutaneous hemangiomas. He was able to sit without support at that time. He is still seen at three-month intervals for out-patient follow-up visits.

## Discussion

Hemangiomas are benign vascular neoplasms, present in nearly 1 to 2% of newborns and 10 to 12% of babies by one year of age. The hemangiomas may occur anywhere on the skin but the head and neck are the most commonly affected sites, followed by the trunk and limbs. In approximately 10 to 20% of babies with hemangiomas, the lesions are multiple [5]. Although the exact mechanism for hemangioma development remains unknown, vascular growth factors seem to play a role in the pathogenesis. Proliferation most probably results from an imbalance between positive and negative angiogenic factors expressed by the hemangioma and adjacent normal tissue [6]. BNH is a nonheritable disorder in which multiple cutaneous capillary hemangiomas appear in an eruptive manner during the neonatal period [7]. BNH was first reported by Stern *et al*. [8]. They increase rapidly in number and size (reaching up to 2cm in diameter) during the first few months and follow a benign course with spontaneous regression, usually within the first four months after their appearance. In general, visceral involvement is absent or unremarkable. Histologically, lesions are typical capillary hemangiomas. Treatment is usually not required because spontaneous resolution occurs with excellent cosmetic appearance [1].

Because benign and diffuse neonatal hemangiomatosis probably exist along a continuum, babies younger than three months with numerous, small cutaneous hemangiomas should be carefully monitored. In this group of patients, in addition to a work-up directed by history, physical examination, general chemistry and blood counts, screening abdominal ultrasonography with Doppler studies may be considered to rule out hepatic involvement [9].

It is well known that thyroid hormones are necessary for growth and development during early infancy and that the early detection and treatment of hypothyroidism is important to prevent growth retardation and intellectual loss [10]. In the first year of life, approximately three to five intelligence quotient (IQ) points are lost for each month in which hypothyroidism remains untreated. This developmentally critical period corresponds to the proliferative phase of hemangiomas and arouses concern that babies with this tumor may be at risk for permanent neurologic damage. Infantile hypothyroidism is often occult, and even severe symptoms could be masked by complications of the hemangioma itself [11].

The aim of treatment of consumptive hypothyroidism due to BNH is to maintain a normal thyroxine level which is vital for the developing brain especially during infancy. This can be achieved by monthly checking of the thyroid function especially during the rapid phase of hemangioma growth then every two months during the stationary and resolution phases [1].

Consumptive hypothyroidism develops when the rate of inactivation of thyroid hormones surpasses the rate of their production, and this may be attributed to the presence of a high level of 3 iodothyronine deiodinase in the hemangioma tissue which catalyzes the conversion of T4 to rT3 and of T3 to diiodothyronine, both of which are biologically inactive [3].

Our index case was diagnosed to have consumptive hypothyroidism secondary to BNH which manifested by developmental delay, puffy eyes, wide anterior fontanelles, cold extremities, hypothermia, bradycardia, abdominal distension, high TSH and rT3 levels, and low FT4 and T3 [12]. Hypothyroidism due to BNH could not be detected on newborn screening as the lesions appeared around the 5th week of life.

The clinical features of both consumptive hypothyroidism due to BNH and simple congenital hypothyroidism are similar, and even some laboratory data are shared by both diseases, for example high TSH and low FT4 and T3 levels. The negative neonatal screening test, late onset of symptoms, normal thyroid scan, presence of multiple skin hemangiomas, high rT3 and the need of a high dose of L-thyroxine treatment are in favor of the diagnosis of consumptive hypothyroidism due to BNH. L-thyroxine in a dose of 5 to 10μg/kg/day is generally adequate for treating congenital hypothyroidism, whereas our index case required higher replacement doses of L-thyroxine (15μg/kg/day) to reduce serum thyrotropin concentrations to normal which are thought to reflect tumor size and type 3 deiodinase activity [3].

## Conclusion

Thyroid function should be assessed periodically in a baby with BNH, especially if symptoms of hypothyroidism appear or the size and number of hemangiomatosis increase rapidly. Moreover, high doses of L-thyroxine may be needed to achieve euthyroidism during the infancy.

## Consent

Written informed consent was obtained from the patient's parents for publication of this case report and any accompanying images. A copy of the written consent is available for review by the Editor-in-Chief of this journal.

## Abbreviations

BNH: Benign neonatal hemangiomatosis;CT: Computed tomography;DNH: Diffuse neonatal hemangiomatosis;FT4: Serum free thyroxine;rT3: Reverse-T3;T3: Triiodothyronine;TSH: Thyroid-stimulating hormone

## Competing interests

The authors declare that they have no competing interests.

## Authors’ contributions

KA and HS diagnosed, investigated, followed up, managed the patient and drafted the manuscript. Both authors read and approved the final manuscript.
